# Breastfeeding training for health professionals and resultant changes in breastfeeding duration

**DOI:** 10.1590/S1516-31802000000600007

**Published:** 2000-11-01

**Authors:** José Augusto de Aguiar Carrazedo Taddei, Marcia Faria Westphal, Sonia Venancio, Cláudia Bogus, Sonia Souza

**Keywords:** Breastfeeding, Developing countries, Hospital practices, Medical education, SLC, Santos Lactation Center, Wellstart, the San Diego Lactation Program, Aleitamento, Países em desenvolvimento, Práticas hospitalares, Educação médica, SLC Centro de Lactação de Santos Wellstart, Programa de Lactação de San Diego

## Abstract

**CONTEXT:**

Promotion of breastfeeding in Brazilian maternity hospitals.

**OBJECTIVE:**

To quantify changes in the breastfeeding duration among mothers served by hospitals exposed to the Wellstart-SLC course, comparing them with changes among mothers attended by institutions not exposed to this course.

**DESIGN:**

Randomized Institutional Trial.

**SETTING:**

The effects of training on breastfeeding duration was assessed in eight Brazilian hospitals assigned at random to either an exposed group (staff attending the Wellstart-SLC course) or a control group.

**SAMPLE:**

For each of the eight study hospitals, two cohorts of about 50 children were visited at home at one and six months after birth. The first cohort (n = 494) was composed of babies born in the month prior to exposure to the Wellstart-SLC course, and the second cohort (n = 476) was composed of babies born six months subsequent to this exposure.

**MAIN MEASUREMENTS:**

Kaplan-Meier curves were plotted to describe the weaning process and log-rank tests were used to assess statistical differences among survival curves. Hazard ratio (HR) estimates were calculated by fitting Cox proportional hazard regression models to the data.

**RESULTS:**

The increases in estimated, adjusted rates for children born in hospitals with trained personnel were 29% (HR = 0.71) and 20% (HR = 0.80) for exclusive and full breastfeeding, respectively. No changes were identified for total breastfeeding.

**CONCLUSION:**

This randomized trial supports a growing body of evidence that training hospital health professionals in breastfeeding promotion and protection results in an increase in breastfeeding duration.

## RESUMO

**CONTEXTO:**

Promoção do aleitamento materno em maternidades brasileiras.

**OBJETIVO:**

Quantificar mudanças na duração do aleitamento materno de mães assistidas em maternidades expostas ao curso Wellstart-SLC, comparando-as com mudanças em mães assistidas por maternidades não expostas.

**TIPO DE ESTUDO:**

Ensaio institucional randomizado.

**LOCAL:**

Os efeitos do treinamento na duração do aleitamento materno foi avaliado em oito maternidades randomicamente alocadas ao grupo exposto (equipe freqüenta o curso Wellstart-SLC) ou controle.

**AMOSTRA:**

Em cada uma das oito maternidades, duas coortes de cerca de 50 crianças foram visitadas em suas casas ao completarem um e seis meses de vida. As primeiras coortes (n = 494) foram compostas de bebês nascidos no mês anterior ao treinamento, enquanto que as segundas coortes (n = 476) foram compostas por bebês nascidos seis meses após a exposição ao curso Weelstart-SLC.

**VARIÁVEIS ESTUDADAS:**

Para descrever o processo de desmame foram traçadas curvas de Kaplan-Meier. Para avaliar as diferenças estatísticas entre as curvas de sobrevivência foi utilizado o teste "logrank". Foram calculadas estimativas das razões de risco(HR) ajustando modelos de regressão de riscos proporcionais de Cox aos dados.

**RESULTADOS:**

O aumento estimado, a partir das razões ajustadas para crianças nascidas em hospitais com pessoal treinado, foi 29% (HR = 0,71) e 20% (HR = 0,80) para aleitamento exclusivo e pleno respectivamente. Não foram identificadas mudanças para o tempo de aleitamento total.

**CONCLUSÕES:**

Esse ensaio randomizado confirma evidências crescentes de que treinar profissionais de saúde em hospitais, na promoção e proteção do aleitamento materno, resulta em aumento do tempo de aleitamento materno.

## INTRODUCTION

Epidemiological studies have identified breastfeeding as a practice that protects children, lowering the risks of disease and death. A review of selected studies has shown that when infants not receiving breast milk are compared to those on exclusive breastfeeding, the median relative risk of diarrhea morbidity ranges from 3.5 to 14.9 at different ages during the first six months of life.^1^ A well-controlled cohort study which included more than 2000 subjects from 33 communities or barangays in metropolitan Cebu, Philippines, found relative risks for diarrhea diseases of 17, 14, and 13 when the weaned children were compared to the exclusively breastfeed ones at ages of 2, 4 and 6 months respectively.^2^ A more recent analysis of the Cebu longitudinal sample including 9,942 children fitted Cox proportional hazard regression models to the data. The adjusted risk ratio for diarrhea mortality in the first six months of life was 9.7 when non-breastfed children were compared to breastfed ones.^3^ A study conducted in Peruvian slums showed that the relative risks of upper and lower respiratory infections among children in their first six months of life are 5.5 and 4.1 respectively for non-breastfeed children, when compared to exclusive breastfed ones.^4^ In a population-based case-controlled study in southern Brazil, premature weaning was found to be an important risk factor for infant deaths from diarrhea and respiratory infection. Compared to infants who were breastfed with no milk supplements, those completely weaned had 14.2 and 3.6 times the risk of death from diarrhea and respiratory infection respectively.^5^

There is some evidence suggesting that breastfeeding can be encouraged by changes in hospital routine and by giving information and support to mothers as well. A review of 21 studies from eight countries shows that the most likely reduction in the prevalence of non-breastfed infants would be: 40% among infants aged 0-2 months, 30% among those aged 3-5 months and 10% among those between 6 months and 1 year old. Theoretical calculations based on these data indicate that breastfeeding promotion can reduce diarrhea morbidity rates by 8 - 20% and diarrhea mortality rates by 24 - 27% in the first six months of life.^6^ Nevertheless, it should be noted that these predictions are based on studies conducted predominantly in developed areas in an effort to assess the effect of various interventions, most of which suffer from methodological problems. Therefore, the reduction estimates should be considered as a best available guess.^7^

The Wellstart Program in San Diego is one of the most promising experiments in breastfeeding promotion. It was created with the objective of training multidisciplinary teams from different regions in lactation management in four-week intensive courses. With their acquired knowledge and techniques, the teams then return to their countries in order to promote changes in routines and procedures aimed at improvement of breast-feeding practices.^8^ As happens with most health activities, the Wellstart experience has not been systematically evaluated. Although participant reports are available and can provide useful insights into the effects of the experience, the information is incomplete and may be affected by significant selection bias.^9^

Brazil is a country where most deliveries occur in hospitals. In the State of São Paulo, the most industrialized state in the country, the hospital delivery rate is higher than 98%. The policies and practices at these hospitals do not encourage breast-feeding: very few have rooming-in and around 30% of the deliveries are cesarean sections. The most recent information on breastfeeding practices in the state, from a survey conducted among children attending the immunization campaign in 1991, showed that the average duration of full breastfeeding was 43 days and that 23% of the children had never been breastfed. As a result, an intervention aimed at the promotion and protection of breastfeeding might have a high impact on infant morbidity and mortality rates.

In April 1990 three professionals from the Santos Lactation Center (SLC), in the State of São Paulo, were trained in the San Diego Center, and in August of the same year similar courses for health professionals were begun in Santos. Since then, four courses have been offered and 120 health professionals have been trained. These courses differ slightly from those in San Diego. They last three weeks instead of four, are conducted in Portuguese, and the preferred candidates are pediatricians, obstetricians and nurses from hospitals, as opposed to universitybased health professionals.

In view of the need to provide a systematic evaluation of the Wellstart-SLC experience, a study was conducted with the following objectives: to identify the positive and negative characteristics of various topics covered in the SLC course and hence improve its effectiveness; to quantify and qualify the structural changes that occurred in hospitals whose staff attended the course by comparing them to similar institutions that had not been exposed; and to quantify changes in breastfeeding duration among mothers served by hospitals exposed to the Wellstart-SLC course by comparing them to changes among mothers attended by institutions not exposed to this course.

A previous publication discussed and evaluated the training course as well as its impact on the implementation of hospital routines favoring breastfeeding, i.e. the first two objectives listed above.^10^ This article presents results concerning changes in breastfeeding duration.

## METHODS

### Study design.

The effect of training on the length of time infants were breastfed was assessed in eight hospitals assigned at random to either the exposed group (staff attending the SLC course) or the control group.^11^ In order to achieve comparability, the eight institutions satisfied the following criteria: public or philanthropic; located near the city of São Paulo, Brazil (within 100 Km); no previous exposure to a similar course; professional staff (two physicians and one nurse) available to attend the course full time for a 3 week period; and at least two births per day in the maternity ward.

For each of the eight study hospitals, two cohorts of about 50 children were visited at home at one and six months after birth. The first cohort was composed of babies born in the month prior to exposure to the SLC course (April 92), and the second cohort was composed of babies born six months subsequent to this exposure (December 92). Twins and newborns with severe diseases that impaired active breastfeeding were excluded. The proportion of losses to follow-up was similar for the before and after course cohorts. Of the original 609 babies that comprised the before-course cohort, 494 (81%) were included in the final analysis, whereas of the 555 original babies that comprised the after-course cohort, 469 (84%) were included in the final analysis. Such losses are likely to have similar direction and intensity in both the exposed and non-exposed cohorts, since they are not related to the process of data collection, but have to do with the characteristics of low-income urban populations. Causes of losses were: family moved to another state, mother intentionally gave wrong address at the time delivery took place, husband abused wife and wife ran away from home, mother was sent to prison, and babies were adopted. Five children died and one mother refused to participate at the six-month visit.

Six questionnaire versions were revised, discussed and pre-tested by members of the research team before deciding on the final versions of the questionnaires for the one and six month postpartum visits. The questionnaires were developed along with a manual that gave some field orientations and directions on how to fill out the forms. Both questionnaires gathered information on the social, economic and demographic characteristics of the family, on the use of health services, on the health conditions of the baby and the mother, on feeding counseling at health services during gestation, delivery and puerperium, and on child and sibling care and feeding practices. Home interviews were carried out by 16 female interviewers who were local residents with high-school diplomas, and who had not attended any health course nor participated in any activity in the area of health. All interviewers took a 25-hour training course and, under supervision, interviewed at least five mothers during the pre-test phase. Eight additional hours of refresher training were given before field surveys began in October 1992 for the six month follow-up on the beforecourse cohort, again in January 1993 for the one month observation on the after-course cohort, and finally in June 1993 for the six month observations on the aftercourse cohort. Ten percent of all interviews were randomly selected and repeated by a field supervisor to check for information reliability.

### Main measurements.

The dependent or effect variable was breastfeeding duration, measured in days, with three components: exclusive breastfeeding (age at introduction of water, tea, or any food other than breast milk), full (age at introduction of any other milk) and total (age at which any breastfeeding was terminated). The independent or exposure variable was whether or not hospital staff had attended the Wellstart-SLC course. Potential confounders or covariates included the mother's age (≤ 19 and >19 years), parity (primiparae and others), kind of delivery (normal and others), birth weight (< and ≥ 2500 g) and sex. Another group of variables that measured changes in hospital procedures related to breastfeeding protection was also taken into consideration. These were five variables regarding whether or not the mother got support for breastfeeding in the hospital, got help for breastfeeding in the hospital, breastfed in the delivery room, breastfed within six hours after delivery, and stayed in a rooming-in facility with her baby.

### Statistical methods.

To assess statistical significance between groups, chi-square tests were used for category variables and t-tests for continuous variables. Kaplan-Meier curves were plotted to describe breastfeeding duration and log-rank tests were used to assess statistical differences among survival curves. The proportional hazards assumption was satisfied since there was no strong evidence of non-parallel- ism of the log-log curves. Hazard ratio (HR) estimates were calculated by fitting Cox proportional hazard regression models to the data using the SAS PHREG procedure. Thirteen regression models were fitted. Six models were for generating crude HR for exclusive, full and total breastfeeding duration, comparing before and after cohorts for controls and exposed hospitals. Another six models were for estimating HR for the same outcome variables and comparing the same cohorts, but adjusted for potential confounding variables. And the last one included before and aftercourse cohorts for exposed and control hospitals to assess the interaction of before and after and controlexposure effects. To assess co-linearity among covariates in the models, an SAS-macro developed by Zack & Rosen was utilized. The probability of erroneously rejecting the null hypothesis (type I error) was established at 0.05 for all statistical inferences.^2-14^

## RESULTS

Table 1 shows that the distributions of the potential confounding variables were not statistically different for the before and after-course cohorts. The typical mother included in the study could be described as young, literate, born in a urban area, having a stable matrimonial relationship with the child's father and willing to breastfeed. Roughly one third were primiparae and two thirds had normal deliveries.

**Table 1:** Comparison of before and after cohorts for exposed and control groups for potential confounding variables

Variables	CONTROL			EXPOSED		
	before	after	P value	before	after	P value
n	239	237		253	241	
% male	48.2	51.8	0.356	47.1	55.9	0.049
Mean birth weight (g)	3184	3194	0.498	3140	3189	0.199
Mean mother's age (years)	28.9	25.8	0.094	26.4	26.0	0.625
% mothers born in urban areas	77	75	0.649	76	77	0.222
% mothers living with fathers	89	86	0.262	82	86	0.146
% literate mothers	94	93	0.843	92	93	0.491
% mothers intending to breastfeed	95	96	0.388	94	90	0.082
% primiparae	44	37	0.252	34	35	0.439
% normal deliveries	65	64	0.583	73	68	0.288

**Figure 1:**
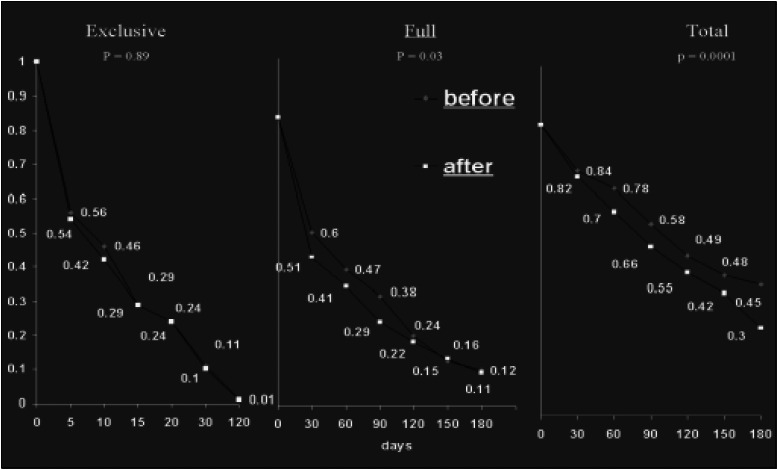
Adjusted* Kaplan-Meier survival curves for breastfeeding duration components (exclusive, full and total), comparing before and after groups for exposed hospitals. *adjusted for hospital of birth, mother's age (≤ 19 and > 19 years), parity (primiparae and others), kind of delivery (normal and others), birth weight (< and ≥ 2500 g) and sex.

**Figure 2:**
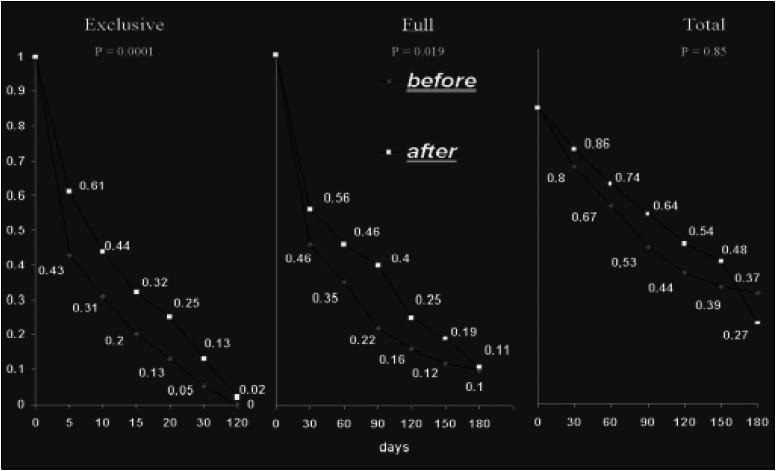
Adjusted* Kaplan-Meier survival curves for breastfeeding duration components (exclusive, full and total), comparing before and after groups for control hospitals. *adjusted for hospital of birth, mother's age (≤ 19 and >19 years), parity (primiparae and others), kind of delivery (normal and others), birth weight (< and ≥ 2500g) and sex.

Figures 1 and 2 display Kaplan-Meyer survival curves comparing breastfeeding duration for the before and after-course cohorts. When children born in the four exposed hospitals before the SLC course took place were compared with children born six months afterwards in the same hospitals, survival probabilities for the three breastfeeding components (exclusive, full and total) were always higher after exposure. The changes were statistically significant for exclusive and full breastfeeding duration among the exposed cohort. When before and after control cohorts were compared, no particular pattern of change could be identified for the exclusive breastfeeding component, while survival curves for full and total breastfeeding duration showed significant decreases.

Table 2 shows that SLC training corresponded to lower rates or velocities in the weaning process for the exclusive and full breastfeeding components. No changes occurred for total breastfeeding among children born in exposed hospitals. For children born in concomitant control hospitals no changes occurred for exclusive breastfeeding, while an increase in the rate of full and total breastfeeding was detected. The results were similar for crude and adjusted hazard ratio estimates for exposed and control groups. Thus, exclusive and full breastfeeding duration became longer among exposed hospitals while full and total breastfeeding duration got shorter among concomitant control hospitals.

**Table 2:** Crude and adjusted hazard ratio (HR) estimates for breastfeeding duration components (exclusive, full and total) of before and after cohorts for exposed and control groups

Breastfeeding Duration Components	CONTROL				EXPOSED				P value interaction*** control-exposed/ before-after
	Crude		Adjusted		Crude		Adjusted		
	HR*	95% CI*	HR	95% CI	HR	95% CI	HR	95% CI	
Exclusive	1.01	0.84-1.21	0.98	0.79 - 1.22	0.69	0.57-0.83	0.71	0.59-0.85	0.0020
Full	1.20	1.00-1.45	1.16	0.93-1.45	0.82	0.68-0.98	0.80	0.67-0.97	0.0019
**Total**	**1.58**	**1.30-1.93**	**1.55**	**1.23-1.96**	**1.02**	**0.84-1.44**	**1.01**	**0.83-1.22**	**0.0019**

* HR = Hazard Ratio estimates; 95% CI = confidence intervals; calculated by fitting proportional hazard Cox regression models.

** adjusted for hospital of birth, mother's age (≤ 19 and >19 years), parity (primiparae and others), kind of delivery (normal and others), birth weight (< and >= 2500g) and sex.;

*** p values for the interaction term of control-exposed and before-after variables from the model that included both cohorts from all hospitals (n=970).

Table 3 shows that there were marked before and after changes among variables related to hospital practices among children born in exposed hospitals, and almost no changes in the same variables for children born in control hospitals. The only significant improvement among children born in control hospitals was the increase in the proportion of children that were breastfed in the delivery room (from 2% to 8%). On the other hand, for exposed hospitals the proportion that were breastfed in the delivery room increased from 2% to 23% while the proportion of newborns that were breastfed in the first six hours of life increased from 41% to 53%. Improvements were also detected in the proportion of mothers that got support and help for breastfeeding their babies from hospital personnel. The proportion of children rooming-in decreased for control hospitals but showed no significant change in exposed hospitals.

**Table 3:** Comparison of before and after cohorts for exposed and control groups for variables related to changes in institutional practices

Variables	CONTROL			EXPOSED		
	before	after	P value	before	after	P value
Frequency	239	237		253	241	
% got support for breastfeeding in hospital	58	61	0.423	48	64	0.000
% got help for breastfeeding in hospital	35	36	0.583	29	49	0.000
% breastfed in delivery room	2	8	0.013	2	23	0.000
% breastfed within six hours	48	50	0.691	41	53	0.009
% roomed-in	20	13	0.029	8	6	0.364

## DISCUSSION

A seasonal bias could have influenced the results, since children included in the before-course cohort were born in April (fall) and the after-course cohort children in December (summer). However, it seems unlikely that the cohorts’ birth months would affect the results since a) seasons are not markedly different in the State of São Paulo, b) most of the mothers lived in urban areas where goods and services are available throughout the year, and c) the concomitant control cohorts showed no changes in hazard ratio estimates for exclusive and full breastfeeding duration and an increase in the hazard ratio estimates for total breastfeeding duration. If, for the sake of argument, a seasonal bias had existed it would have had the effect of hiding a significant decrease in hazard ratio estimates for total breastfeeding duration, since the expected change with no exposure would have been an increase in this estimate.

Another important point to consider is whether the observed changes are permanent or temporary. According to our findings, modifications in hospital practices that were significant were related to staff behavior rather than to structural changes. For instance, no changes were identified for rooming-in, a physical change, while all the other variables presented in Table 3, dependent upon personnel practices, resulted in significant changes. Results from the qualitative component of this trial suggest that followup support should be provided for the trained team in order to promote permanent institutional changes.^10^ Follow-up activities should include a critical analysis of the institutional changes required for establishing an effective breastfeeding protection and promotion program. Success would depend on better cohesion between the institutional domains for policy, management and services.^15^

## CONCLUSION

This randomized trial supports a growing body of evidence that training hospital health professionals in breastfeeding promotion and protection results in an increase in breastfeeding duration. The increases in estimated, adjusted hazard ratios for children born in hospitals with trained personnel were 29% (HR = 0.71) and 20% (HR = 0.80) for the exclusive and full breastfeeding (Table 2), respectively. No changes were identified for total breastfeeding, probably because interventions that take place only at birth have less of an influence on mothers’ feeding practices as the child gets older.
